# Supplementary material for: Rhesus macaques model human Mayaro virus disease and transmit to Aedes aegypti mosquitoes
Source: PLoS Negl Trop Dis. 2025 Oct 29;19(10):e0013061. doi: 10.1371/journal.pntd.0013061 (PMC12582505; doi:10.1371/journal.pntd.0013061)
Supplement: S1 Table — (DOCX) [file pntd.0013061.s003.docx]

| Score* | Description | Example |
| --- | --- | --- |
| 0 | No inflammation = Within normal histological parameters with no inflammatory cells observed | 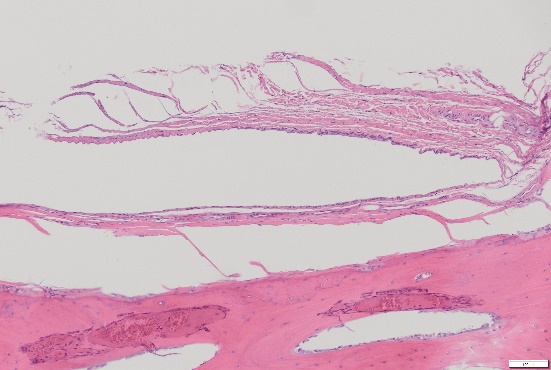 |
| 1 | Minimal inflammation = Few scattered individual inflammatory cells (predominately lymphocytes) or aggregates of less than 5 within the synovium and/or joint capsule. | 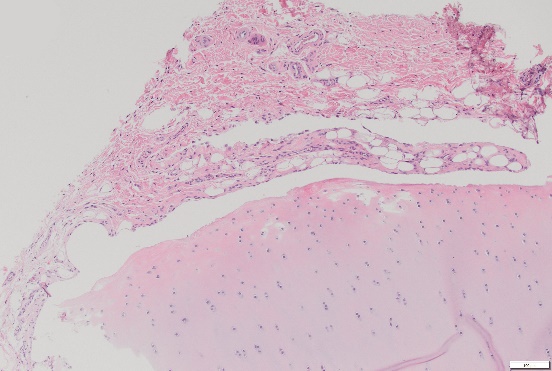 |
| 2 | Mild inflammation = Inflammation (predominately lymphocytes) forms small aggregates of 5-20 cells per aggregate within the synovium and/or joint capsule. | 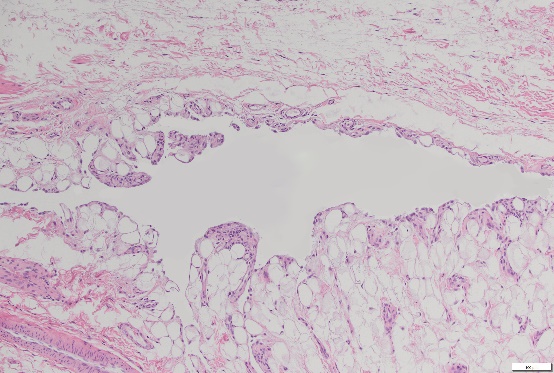 |
| 3 | Moderate inflammation = Inflammatory cells (predominately lymphocytes) form aggregates of more than 20 cells per aggregate within the synovium and/or joint capsule. | 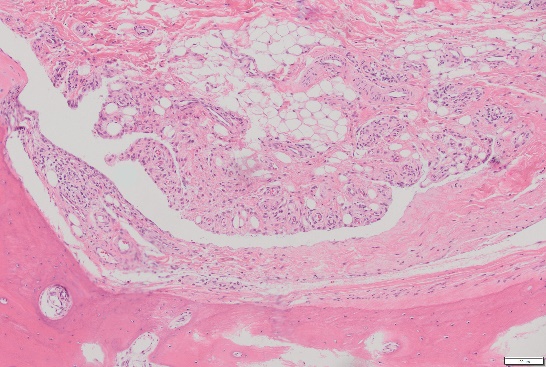 |
|  | * = Add 1 point for presence of fibrin within joint | 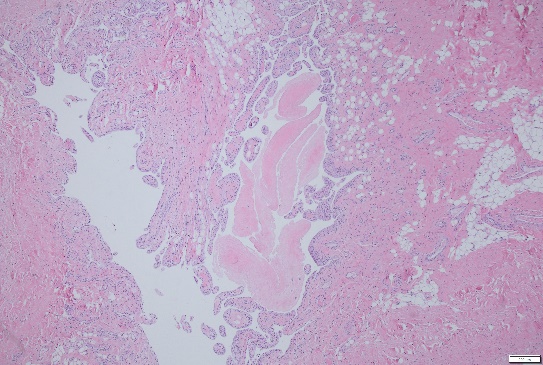 |
