# Supplementary material for: Rhesus macaques model human Mayaro virus disease and transmit to Aedes aegypti mosquitoes
Source: PLoS Negl Trop Dis. 2025 Oct 29;19(10):e0013061. doi: 10.1371/journal.pntd.0013061 (PMC12582505; doi:10.1371/journal.pntd.0013061)
Supplement: S2 Table — Primers were designed to match the genome of the parent strain, GenBank: MK070491. (DOCX) [file pntd.0013061.s004.docx]

| **Genome position** | |  |
| --- | --- | --- |
| **Start** | **End** |  |
| 63 | 90 | AAGACTTTCGACATTCTGAGTGGTAAAC |
| 246 | 269 | CGATGTCCAGGATGAGTGTGTCT |
| 415 | 438 | GACTGCAGGTCGTCTATCTTTGC |
| 596 | 619 | GGTAGTGTCGAACCCAATCCAGT |
| 780 | 803 | CCGCGAACATTATCTGGTCACTC |
| 957 | 981 | CCTGACGTTTTTCCGAATACCCCT |
| 1102 | 1122 | TCAGTCGCCAGAATGCCTGT |
| 1290 | 1313 | GTTCGCGCATACCCATGTTTTTC |
| 1478 | 1502 | GTCTTGACTGCAAAAGAAGCCTGA |
| 1640 | 1664 | GCTCCTCTACATCAACTTCAGGGA |
| 1779 | 1806 | AGTACCTGCAGTTTAACACTTTTCAGG |
| 1963 | 1988 | TGCGATTGATAAACTCACGTTCGTT |
| 2137 | 2159 | GGTTGACCAGGTCTCCGATCAT |
| 2321 | 2343 | TCGTGCATGATCTCCTGACAGT |
| 2492 | 2516 | AAAGCACAACCTTCTTACGTGGTC |
| 2679 | 2708 | TGATAACGGGTTTATTGTATGGGTTAGTG |
| 2864 | 2889 | AGTGGGTTTTCGTTGACCTTCATTC |
| 3028 | 3050 | CGTCGTGCTCTTGTTGCCAATC |
| 3213 | 3237 | AGTGCAACCTCTGGTGAATAAGCT |
| 3342 | 3365 | AAAACCCGTACATCCTTCCTCCA |
| 3525 | 3554 | CCACTCTTTCTCCCTTAACTGGATGATAT |
| 3680 | 3705 | TTAGGCGGTAGTCCTAAATCCAAGT |
| 3816 | 3838 | CCCGGGTTTCAACAGGTAGAGT |
| 3990 | 4015 | CAGGGTTACTGTTCTTCTACCGTTG |
| 4143 | 4164 | ACTTGTCCACGGTGGTTAGCT |
| 4305 | 4327 | ACGATCCCCTTCAGCCTCAGAT |
| 4485 | 4507 | GCAGTAGATGGTAACCCGTGCT |
| 4664 | 4699 | GTGGAACTTAGTACCTTCCATATATGAATATAAGG |
| 4823 | 4848 | GGTGGAGTCGATGAGTCACTATCCT |
| 5000 | 5025 | GGTGGAGTTGCATCAAAAAGCATCA |
| 5155 | 5178 | CGCATGGACTGTGGATCATTACC |
| 5324 | 5344 | TGGGACCGGTTTCGTCCTTT |
| 5426 | 5448 | CCGAACTCTATGTCAACGGCCT |
| 5595 | 5618 | CCTCTAGGTCGTGTTGTCTCACT |
| 5754 | 5783 | GGTCAATGATTGTTGCTTTTATGTTCTCC |
| 5927 | 5949 | GGGTAGTTCCGGGCCAGAAATT |
| 6103 | 6125 | TTTGGAATGGCGAAGGAACAGC |
| 6267 | 6297 | GTTGTAATTCTGATAGGTTTAGCAGCAAAC |
| 6452 | 6474 | ACCTTTGGTCGTTCCTCTGTGT |
| 6598 | 6627 | TGCTCTGATATGATGGCATCAAAATCTTC |
| 6774 | 6796 | CAGGTGGCAGCTGGTAATCTGA |
| 6931 | 6953 | CGTTGTCGTCGCCGATAAATGC |
| 7096 | 7117 | TGCTATTCGACACGACGTCCT |
| 7219 | 7241 | CCGAACCCAGGCCTGATCTAAA |
| 7357 | 7380 | TTAGGACCGCCGTACAAGTGTAT |
| 7526 | 7546 | CGGCAATCAGCTGCTGCATT |
| 7709 | 7732 | GCTCAATCTTCATGCACATGCGC |
| 7893 | 7915 | TCATAGCCACCGGTATCTGTGC |
| 8046 | 8068 | GGCCCTTGTTGTCAAAGATGGG |
| 8215 | 8238 | ACAAGGGAAGGATACATTCGCCA |
| 8348 | 8371 | TCGAGCTGTTTTTGCAGTACACG |
| 8520 | 8547 | GCCAATTTGAGAAGCGAACTGAATTTT |
| 8698 | 8721 | GACACTAATGACTTCGCCTGGTG |
| 8841 | 8863 | CGGTCGTTAGCTGGTAAGTGGT |
| 9029 | 9054 | TTTGTCAACGGTGCAGCTATTGATA |
| 9206 | 9231 | CGTAGCTTCTCGTTTACCGCTTCTA |
| 9375 | 9397 | ACCACAAGCGTTGCGGTTTATG |
| 9545 | 9566 | CACTTGTTGCGTGCCACTACA |
| 9726 | 9753 | GCAACATGTAGTCAGGATAAGAAGAGC |
| 9883 | 9905 | GGGCTGTATCCTTCACGTGCAA |
| 10051 | 10074 | CGTGAACACCGCACACTTGTATT |
| 10209 | 10232 | ACCTTGATTTTGGCTCTGAGGGA |
| 10383 | 10405 | CATACGGGGGAAAGTCCTGGTT |
| 10553 | 10577 | GGTGAGTCCCTGTCTTTTTGCCAT |
| 10729 | 10752 | ATGCGTGCATGTAGATACAGTGC |
| 10910 | 10935 | GCTGCAGACGGATACTATGAAGGAA |
| 11096 | 11132 | CTCAAAGTTATGCAAGTAACTATTACTAGAATTAGC |
| 11242 | 11273 | GGGTTACATATGTGAATAATAAATCGGTCCG |
