# Supplementary material for: Rhesus macaques model human Mayaro virus disease and transmit to Aedes aegypti mosquitoes
Source: PLoS Negl Trop Dis. 2025 Oct 29;19(10):e0013061. doi: 10.1371/journal.pntd.0013061 (PMC12582505; doi:10.1371/journal.pntd.0013061)
Supplement: S3 Table — Inoc. is inoculation, DPI is day post-inoculation, PFU is plaque forming unit, IV is intravenous, SC is subcutaneous. (DOCX) [file pntd.0013061.s005.docx]

| **Observation** | **DPI** | **Inoc. Route** | **Dose (log _10_ PFU)** | **Mosquito Feed** |
| --- | --- | --- | --- | --- |
| none | 0 | IV | 7 | No |
| none | 1 |  |  | No |
| bruising at leg injection site | 2 |  |  | Yes |
| mild inguinal rash | 3 |  |  | Yes |
| mild redness at injection site | 4 |  |  | No |
| mild redness at feeding site | 5 |  |  | Yes |
| mild rash at feeding site | 6 |  |  | No |
| no rash, abdominal pigment change that is cold to touch | 7 |  |  | Yes |
|  |  |  |  |  |
| none | 0 | IV | 7 | No |
| none | 1 |  |  | No |
| none | 2 |  |  | Yes |
| none | 3 |  |  | Yes |
| abdominal redness from mosquito bites | 4 |  |  | No |
| mild redness at feeding site | 5 |  |  | Yes |
| redness at feeding site | 6 |  |  | No |
| none | 7 |  |  | Yes |
| none | 10 |  |  | No |
| none | 12 |  |  | No |
|  |  |  |  |  |
| none | 0 | SC | 7 | No |
| none | 1 |  |  | No |
| none | 2 |  |  | Yes |
| abdominal bumps from mosquito feeding | 3 |  |  | Yes |
| mild redness between shoulders | 4 |  |  | No |
| mild redness at feeding site | 5 |  |  | Yes |
| redness at feeding site | 6 |  |  | No |
| mild rash on abdomen of mosquito feeding site | 7 |  |  | Yes |
| none | 10 |  |  | No |
| none | 12 |  |  | No |
|  |  |  |  |  |
| none | 0 | SC | 7 | No |
| none | 1 |  |  | No |
| none | 2 |  |  | Yes |
| razor burn on abdomen | 3 |  |  | Yes |
| none | 4 |  |  | No |
| scratching at right thigh | 5 |  |  | Yes |
| redness at feeding site | 6 |  |  | No |
| mild rash on abdomen of mosquito feeding site | 7 |  |  | Yes |
|  |  |  |  |  |
| none | 0 | IV | 7 | No |
| none | 1 |  |  | No |
| bruising at leg injection site | 2 |  |  | Yes |
| none | 3 |  |  | Yes |
| None | 4 |  |  | No |
| none | 5 |  |  | Yes |
| mild abdominal rash from mosquito feeding | 6 |  |  | No |
| none | 7 |  |  | No |
| mild abdominal rash | 10 |  |  | No |
|  |  |  |  |  |
| minor bruise between shoulder blades | 0 | SC | 3 | No |
| none | 1 |  |  | No |
| mild rash-redness at leg injection site | 2 |  |  | Yes |
| right shoulder mild scratch-redness | 3 |  |  | Yes |
| none | 4 |  |  | No |
| none | 5 |  |  | Yes |
| mild inguinal rash/redness | 6 |  |  | No |
| none | 7 |  |  | No |
| none | 10 |  |  | No |
|  |  |  |  |  |
| two small red spots on ribs | 0 | SC | 3 | No |
| none | 1 |  |  | No |
| mild abdominal rash | 2 |  |  | Yes |
| mild abdominal rash - mosquito bites | 3 |  |  | Yes |
| mild abdominal rash | 4 |  |  | No |
| mild abdominal rash | 5 |  |  | Yes |
| redness between shoulder blades; abdominal rash and redness | 6 |  |  | No |
| left leg injection site rash and bruising | 7 |  |  | No |
| none | 10 |  |  | No |
| none | 12 |  |  | No |
|  |  |  |  |  |
| none | 0 | SC | 7 | No |
| none | 1 |  |  | No |
| moderate abdominal rash | 2 |  |  | Yes |
| moderate abdominal rash | 3 |  |  | Yes |
| mild abdominal rash not associated with feeding (pre experimental) | 4 |  |  | No |
| mild abdominal rash not associated with feeding | 5 |  |  | Yes |
| mild abdominal rash from mosquito feedings | 6 |  |  | No |
| none | 7 |  |  | No |
| mild abdominal rash | 10 |  |  | No |
| red lines along left thigh | 12 |  |  | No |
|  |  |  |  |  |
| none | 0 | SC | 7 | No |
| mild redness between shoulder blades | 1 |  |  | No |
| redness at injection site; left arm rash | 2 |  |  | Yes |
| mild rash on abdomen | 3 |  |  | Yes |
| none | 4 |  |  | No |
| mild rash on abdomen and between shoulder blades | 5 |  |  | No |
| moderate rash and scratch marks on abdomen | 6 |  |  | No |
| none | 10 |  |  | No |
|  |  |  |  |  |
| none | 0 | IV | 7 | No |
| none | 1 |  |  | No |
| none | 2 |  |  | Yes |
| none | 3 |  |  | Yes |
| none | 4 |  |  | No |
| none | 5 |  |  | No |
| none | 6 |  |  | No |
| none | 10 |  |  | No |
| none | 12 |  |  | No |
|  |  |  |  |  |
| none | 0 | SC | 3 | No |
| mild redness between shoulder blades | 1 |  |  | No |
| medial thigh red lesion | 2 |  |  | Yes |
| none | 3 |  |  | Yes |
| none | 4 |  |  | No |
| mild redness between shoulder blades | 5 |  |  | No |
| mild redness between shoulder blades | 6 |  |  | No |
| none | 10 |  |  | No |
|  |  |  |  |  |
| none | 0 | SC | 3 | No |
| mild redness on left leg shaved area | 1 |  |  | No |
| none | 2 |  |  | Yes |
| mild rash on abdomen and testicles | 3 |  |  | Yes |
| mild rash on abdomen and cheek | 4 |  |  | No |
| none | 5 |  |  | No |
| mild rash on back of left leg | 6 |  |  | No |
| rash at mosquito feeding site | 10 |  |  | No |
| mild rash around mouth | 12 |  |  | No |
